# Sex-Differences, Handedness, and Lateralization in the Iowa Gambling Task

**DOI:** 10.3389/fpsyg.2016.00708

**Published:** 2016-05-31

**Authors:** Varsha Singh

**Affiliations:** Humanities and Social Science, Indian Institute of Technology DelhiNew Delhi, India

**Keywords:** decision-making, handedness, Iowa Gambling Task, laterality, reward punishment, task motivation

## Abstract

In a widely used decision-making task, the Iowa Gambling Task (IGT), male performance is observed to be superior to that of females, and is attributed to right lateralization (i.e., right hemispheric dominance). It is as yet unknown whether sex-differences in affect and motor lateralization have implications for sex-specific lateralization in the IGT, and specifically, whether sex-difference in performance in the IGT changes with right-handedness or with affect lateralization (decision valence, and valence-directed motivation). The present study (*N* = 320; 160 males) examined the effects of right-handedness (right-handedness vs. non-right-handedness) as a measure of motor lateralization, decision valence (reward vs. punishment IGT), and valence-directedness of task motivation (valence-directed vs. non-directed instructions), as measures of affective lateralization on IGT decision making. Analyses of variance revealed that both male and female participants showed valence-induced inconsistencies in advantageous decision-making; however, right-handed females made more disadvantageous decisions in a reward IGT. These results suggest that IGT decision-making may be largely right-lateralized in right-handed males, and show that sex and lateralized differences (motor and affect) have implications for sex-differences in IGT decision-making. Implications of the results are discussed with reference to lateralization and sex-differences in cognition.

## Introduction

The Iowa Gambling Task (IGT: Bechara et al., 1994) is a widely used neuropsychological decision-making task that offers a choice between immediate vs. long-term gains. The task has been useful in addressing important theoretical issues pertaining to decision neuroscience, for example, the role of working memory and executive function (Bechara et al., 1998; Turnbull et al., 2005), and the nature of insight—implicit or explicit—into the reinforcement (Maia and McClelland, 2004; Bechara et al., 2005). The task has also been instrumental in understanding the role of the prefrontal cortex (PFC) and sub-regions (e.g., dorsal vs. ventral regions of the PFC; Fellows and Farah, 2005). It has been observed that more males than females make advantageous decisions in this task (Reavis and Overman, 2001; Bolla et al., 2004; van den Bos et al., 2007; see review by van den Bos et al., 2013), and that the right hemisphere seems to be more involved than the left in advantageous decision-making (e.g., Manes et al., 2002; Tranel et al., 2002; Clark et al., 2003; Buelow and Suhr, 2009). Even though sex differences may emerge in the IGT because it seems to be primarily right-hemisphere task (Bolla et al., 2004), it remains unclear whether sex and lateralization contribute to IGT decision-making (where lateralization is defined as asymmetrical engagement of the two hemispheres of the brain).

Of the three commonly used neuropsychological decision-making tasks (i.e., the IGT, the Cambridge risk task, and the Risk task), the IGT alone shows lateralization (Clark et al., 2003). Decision-making in the IGT has primarily been associated with the right hemisphere (Naccache et al., 2005; Christman et al., 2007). For instance, research on unilateral lesions suggests that functioning of the right hemisphere is largely crucial to decision-making in the IGT (Tranel et al., 2002) because IGT decision-making seems to show greater impairment for right- vs. left-lateralized lesions in the PFC (e.g., Manes et al., 2002; Bark et al., 2005), with lesion size correlating with disadvantageous IGT choices (Clark et al., 2003). However, lesion studies are fraught with problems, such as the absence of strictly lateralized damage, lack of specificity in the lateralization of the damage, and the small numbers of patients with appropriate lesions (Fellows and Farah, 2005). Additionally, brain activation studies rarely indicate that functions are governed by hemispheres on an absolute “all or none” basis; rather, a functional lateralization approach suggests that a function evokes asymmetrical or a graded activation across the two hemispheres (Knecht et al., 2000). Furthermore, limitation of applying the lateralization approach to explain complex tasks, i.e., tasks in which multiple constructs rather than a unitary construct drive performance, should be acknowledged; thus, lateralization might partially (rather than completely) explain sex-differences in decision-making (Rilea et al., 2004). Nevertheless, discounting sex differences in neuroscience, including in studies involving diagnostic tools, such as the IGT, could result in an incomplete understanding of brain and behavior, and psychological disorders (Cahill, 2006). Researchers have observed that consistent sex differences in widely used tasks should be re-examined to understand social issues, such as the link between sex differences in cognitive processing and the under-representation of females in science and engineering (Miller and Halpern, 2014). The present paper aims to understand sex-differences in the IGT using a functional lateralization approach.

It has been observed that there are sex-differences in the extent to which a function asymmetrically implicates a hemisphere. For example, males tend to show greater lateralization of functions compared to females (Inglis and Lawson, 1981; Azari et al., 1995; Bolla et al., 2004). Specifically, language seems to be more strongly left-lateralized in males than in females (Shaywitz et al., 1995), and performance on emotional-face processing tasks is more strongly right-lateralized in males than in females (Bourne, 2005). A recent review addressing sex-differences in the IGT noted that IGT decision-making may be predominantly right-lateralized in males and left-lateralized in females (van den Bos et al., 2013). In fact, advantageous decision-making in the IGT reflects cognitive control wherein a reflective system over-rides the impulse to choose immediate rewards, and guides long term advantageous decision-making (Bechara, 2005), and some studies have observed that cognitive control is largely right-lateralized (Garavan et al., 1999; Aron et al., 2003, 2004; Knoch et al., 2006). Further, right-lateralization of cognitive control seems to differ between sexes; for instance, due to the distinct organization of inter-hemispheric interactions (specifically the morphology of the corpus callosum), males tend to show greater functional lateralization of cognitive control than females (Huster et al., 2011). Compared to other cognitive control tasks (e.g., the Stroop task), the IGT shows the most prominent sex-differences in lateralization, whereby males primarily show right hemispheric activation whereas females show more activation predominantly in the left hemisphere (Bolla et al., 2004). Observations from lesion studies also suggest that there are sex and laterality differences in the IGT. For instance, the originators of the IGT (Tranel et al., 2005) compared four males and four females, each with a unilateral lesion on either the left or the right side, and found that right-hemisphere damage leads to decision-making deficits in male patients, whereas damage to the left hemisphere is detrimental in this respect in female patients. Therefore, lateralized activation observed via brain imaging studies as well as IGT deficits observed in unilateral lesion studies suggest that the lateralization of IGT-related decision-making is sex-specific.

Furthermore, cognitive control in IGT decision making seems to be sensitive to punishment, suggesting that affect lateralization, that is, right lateralization of negative emotion and avoidance motivation, and left lateralization of positive emotion and approach motivation (Davidson, 1992, 1995, 2004) might influence IGT decision making. A punishment variant of the IGT was introduced by the originators of the IGT, in which participants are required to choose between high losses and high gains vs. low losses and low gains; the choice of high immediate losses/high long-term gains reflects decision-making that is advantageous in the long-term (see Table 1). It was expected that healthy normal participants would make advantageous decisions in both decision frames, irrespective of the frame of the decisions, i.e., whether the decision presented was in a “gain” frame of foregoing immediate reward in the reward IGT, or in a “loss” frame of bearing immediate losses in the punishment IGT (Bechara et al., 2000). However, more advantageous IGT decision-making is observed in the punishment IGT than in the reward IGT (e.g., Must et al., 2006, 2007; Verdejo-Garcia et al., 2007), which suggests that the punishment IGT may be conducive to cognitive control. Since few studies use both reward and punishment IGT tasks, it is unclear whether sex-differences in affect lateralization will influence difference in advantageous decision-making across reward and punishment IGT.

**Table 1:** **Characteristics of reward IGT (decks A′, B′, C′, and D′) and punishment IGT (decks E′, F′, G′, and H′) (Bechara et al., 2000)**.

**Reward IGT**	**Deck A′ (Risky)**	**Deck B′ (Risky)**	**Deck C′ (Safe)**	**Deck D′ (Safe)**
	High immediate reward— High long term loss	High immediate reward— High long term loss	Modest immediate reward— Modest long term loss	Modest immediate reward— Modest long term loss
**Punishment IGT**	**Deck E**′ **(Safe)**	**Deck F**′ **(Risky)**	**Deck G**′ **(Safe)**	**Deck H**′ **(Risky)**
	High immediate losses— High long term rewards	Low immediate losses— Low long term rewards	High immediate losses— Low long term rewards	Low immediate losses— Low long term rewards

Previously, it was observed that the instruction to seek reward rather than the bi-directional instruction to seek reward and avoid punishment contributed to a difference in advantageous decision-making in the two IGTs (Singh and Khan, 2012), and facilitated differentiation between long-term and frequency-based decision-making in the reward IGT (Singh, 2013). This suggested that valence-directedness of task motivation, that is, motivation directed toward either reward or punishment, rather than directed toward both reward and punishment, improves advantageous decision-making in the IGT, possibly due to reduced cognitive processing demands (Singh and Khan, 2012; Singh, 2013). It is also possible that valence-directed motivation triggers much more lateralized activity than (a) motivation directed toward both reward and punishment, (which might trigger more bilateral activity), and (b) motivation that is neither directed toward reward nor punishment, (which might trigger less lateralized activity). Thus, the valence-directedness of task motivation could generate strong lateralization and might reveal sex-differences. It has been observed that the observed male advantage in the reward IGT is due to greater punishment sensitivity. However, the female disadvantage has been attributed to either greater focus on rewards (Bolla et al., 2004; Evans and Hampson, 2015) or undifferentiated attention toward both reward and punishment (Stout et al., 2005). It is possible that valence-directedness in males triggers lateralized activity, which is conducive to advantageous IGT decision-making, whereas undifferentiated focus on rewards and punishments in females might trigger bilateral activity, which is not conducive to cognitive control.

Furthermore, motor laterality, specifically individual differences in right-handedness, could have implications for sex-specific lateralization in the IGT, because sex differences in handedness reflect sex-dependent differences in cerebral organization, which has implications for cognitive functions. For instance, language seems to be strongly left-lateralized in right-handers (Carey and Johnstone, 2014), whereas females seem to show less language lateralization, irrespective of handedness (Hagmann et al., 2006). Similarly, affect lateralization seems sex- and handedness-specific: affect lateralization is observed for right-handedness, but not for left-handedness (Brookshire and Casasanto, 2012), and is more pronounced in males than in females (Wager et al., 2003), reversing with the direction of handedness. Processing of facial emotion is strongly right-lateralized in right-handed males, whereas the relationship between right-handedness and lateralization of facial emotion processing for females is weak or non-existent (Bourne, 2008). Right-handedness influences sex-differences particularly for right-lateralized tasks (Crucian and Berenbaum, 1998). It has been reported that strongly right-handed individuals have less interhemisheric interaction and restricted access to the right hemisphere compared to mixed or non-right-handed individuals (Christman et al., 2004; Propper et al., 2005). It is possible that restricted right-hemispheric access among strong right-handers will influence their IGT-related decision-making and that the effect of restricted right-hemisphere access will differ between the sexes. In other words, strong right-handedness will have implications for sex-specific lateralization of IGT decision-making, particularly if the right hemisphere is critical for IGT-related decision-making.

The present study examined the relationship between sex, and motor and affective lateralization and its effect on cognitive control in IGT decision-making. It was expected that sex and lateralized differences in motor, affect, and cognitive control would have implications for sex-differences in advantageous IGT decision-making. Cognitive control reflected in advantageous decision-making was expected to alter across reward and punishment IGT variants, and this change was expected to differ according to sex (male vs. female), right-handedness, (right-handed vs. non-right-handed), and the valence-directedness of task instructions (directed vs. non-directed). It was expected that there would be an interaction between cognitive control and sex, as well as motor and affective lateralization. That is, lateralization of motor, affect, and cognitive control was expected to drive sex-differences in IGT-related decision-making.

## Materials and methods

### Participants

Three-hundred-and-twenty healthy and medication-free students (mean age = 23.81 years, SD = 3.24; 160 male) volunteered to participate in this study. The experiment was conducted in accordance with the Declaration of Helsinki; all participants provided informed consent, and the study was approved by the Research Committee of the Indian Institute of Technology–Bombay where the research was conducted.

### Design and variables

The design consisted of two levels of total net scores as a within-participant factor (reward/punishment IGT) × 2 types of task motivation (valence-directed/non-directed) × 2 levels of right-handedness (right-handed/non-right-handed) as between-participant factors. All participants performed both variants of the task (reward and punishment variants, in a counterbalanced order). Half of the sample (*n* = 160) received valence-directed instructions (i.e., reward-directed [*n* = 80] or punishment-directed [*n* = 80]). The other half received valence non-directed instructions (*n* = 160; i.e., both reward and punishment [*n* = 80] or no suggestions regarding reward or punishment [*n* = 80]). All participants performed both variants (reward variant and punishment variant) for one instruction type, and the IGT scores on the two IGT variants served as a within-participant factor.

Cards drawn from each of 4 decks of the reward IGT and from each of the decks of punishment IGT served as a variable for deck-wise analysis. For block-wise analysis, in each of 5 blocks of 20 trials, the number of times a deck was chosen during the block of trials was calculated (i.e., for decks A′ through D′ in the reward variant) to produce block-wise net scores, and a total net score for the reward variant using the following formula: (C′ + D′) − (A′ + B′). Similarly, in the punishment variant, the total net score was calculated as (E′ + G′) − (F′ + H′).

### Procedure

The Edinburgh Handedness Inventory (Oldfield, 1971) was used to determine right-handedness, wherein the inventory score ranges from −100 (left-handedness) to 100 (right-handedness) and a score less than 0 is considered to indicate left-handedness. The current sample had a median score of 80, scores above 80 reflected strong right-handedness (*n* = 144); 80 is also the population median score obtained in the original study using a large database (Oldfield, 1971). Handedness is considered as a continuous variable; however, studies that test differences between groups use the population median score of 80 (e.g., Christman et al., 2007; Westfall et al., 2010; Lyle and Orsborn, 2011; Westfall et al., 2014). In fact, the inclusion criterion for right-handed participants in lateralization studies is a cut-off of 30; thus, anyone scoring above 30 is considered a right-hander (e.g., Knecht et al., 2000). Groups made on the basis of the median combines mixed and left handers into a group of non-right-handers, which allows a comparison between right-handedness and non-right-handedness, rather than simply comparing right- vs. left-handers; the latter is no longer considered as a the only robust classification of handedness (Prichard et al., 2013).

Computerized versions of the reward IGT and punishment IGT were used (Bechara et al., 1994). The IGTs were presented in a counterbalanced design with a 5-min break between the 2 variants. Task instructions were administered before presenting the task. Task motivation was manipulated via the task instructions, such that valence-directed instructions urged the participant toward either seeking rewards or avoiding punishments (*n* = 160). In contrast, non-directed instructions lacked valence-directedness (*n* = 160). The instructions are provided in the Appendix.

### Data analysis

All analyses were completed using the Statistical Package for the Social Sciences (SPSS 16, India), with the level of significance set to 0.05, and the data were split by sex. Decision-making in the IGT is commonly analyzed using the “net score” method, wherein the deck choices are aggregated; that is, the total cards drawn from the 2 bad decks are deducted from the total cards drawn from the 2 good decks. The net score method has been criticized (Lin et al., 2007); however, to enable a comparison between the present results and the previous findings on sex-differences in the IGT (e.g., Bolla et al., 2004), particularly when the comparison is cross-cultural (e.g., American- Brazil comparison: Bakos et al., 2010), it was crucial to retain the net score method for analyzing IGT decision-making, additionally, IGT decision-making is also analyzed using individual decks and blocks of trials. Correlations were used to determine whether handedness as a continuous variable is associated with IGT net scores in the reward and the punishment IGT, after partialling out the effects of sex and task instructions. Mixed ANOVA were used on decks (number of cards drawn from individual decks) and blocks (net scores on a block of 20 trials). Decision-making in the IGT was analyzed separately for reward and for punishment IGT, followed by comparison of advantageous decision-making across the 2 IGTs, wherein net scores on the 2 IGTs were considered the within-participants factor (scores consisted of [decks C′ + D′] − [decks A′ + B′]) and ([E′ + G′] − [F′ + H′]), and handedness (right-handed vs. non-right-handed) and instruction type (valence-directed vs. non-directed) were considered the between-participants factors. A Huynh–Feldt correction for epsilon values greater than 0.75 was used. Box's test was used to show that the data did not violate the assumption of equality of covariance matrices.

### Results

Table 2 shows descriptive statistics of the sample set. There was no significant correlation between handedness as a continuous variable and net scores on reward and punishment IGT. Furthermore, partial correlation, wherein the effects of sex were controlled for, between handedness and both reward IGT and punishment IGT net scores failed to reach statistical significance. However, partial correlation controlling for sex and valence-directed task instructions showed that right-handedness was positively correlated with advantageous decision-making in the punishment IGT (*r* = 0.10; *p* < 0.05; see Table 3). As expected, right-handedness was associated with advantageous decision-making in the punishment IGT variant, and sex and task motivation appeared to be critical to the right-handed advantage in the punishment IGT variant.

**Table 2:** **Descriptive statistics of the sample (***N*** = 320, male = 160)**.

**Sex**	**Handedness**	**Instructions**	***N***
Male	RH	Valence-directed	26
		Non-directed	35
	NRH	Valence-directed	54
		Non-directed	45
Female	RH	Valence-directed	41
		Non-directed	42
	NRH	Valence-directed	39
		Non-directed	38

“*RH” denotes right-handedness and “NRH” denotes non-right-handedness*.

**Table 3:** **Correlation between handedness and net scores on the reward and punishment IGT, taking into account the effect of sex and valence-directed instructions**.

		**Edinburg laterality quotient**	**Net reward IGT**	**Net punishment IGT**
Simple correlation (*N* = 320)	Edinburg	1	−0.02 (0.38)	0.07 (0.09)
	Reward	−0.02 (0.38)	1	0.07 (0.10)
	Punishment	0.07 (0.09)	0.07 (0.10)	1
Controlling for sex (*df* = 317)	Edinburg	1	−0.01 (0.44)	0.09 (0.06)
	Reward	−0.01 (0.44)	1	0.07 (0.11)
	Punishment	0.09 (0.06)	0.07 (0.11)	1
Sex and instructions (*df* = 316)	Edinburg	1	0.00 (0.48)	0.10 (0.04)^*^
	Reward	0.00 (0.48)	1	0.05 (0.18)
	Punishment	0.10 (0.04)^*^	0.05 (0.18)	1

The results of 3 correlations (simple and partial) between scores on the Edinburg Inventory of Handedness and net scores on the reward IGT and the punishment IGT attained significance when the effects of sex and instructions were partialled out (

“*” *, significance level of 0.05). Values denote correlation and one-tailed level of significance in the bracket*.

Handedness, sex, and task instruction were correlated with advantageous decision-making in the punishment IGT but male advantage in IGT decision making remains to be addressed. To address the male-advantage observed in the IGT reward variant in the literature, and to ascertain whether laterality contributes to sex-differences in IGT the sample was split on the basis of sex (male vs. female), motor laterality or right-handedness (right-handed vs. non-right-handed), affect laterality or valence-directedness of task motivation (valence-directed vs. non directed), and advantageous decision-making in the reward and punishment IGT were compared using 6 mixed ANOVAs (2 ANOVAs for deck analysis and 4 ANOVAs for net score analysis).

**Figure 1:**
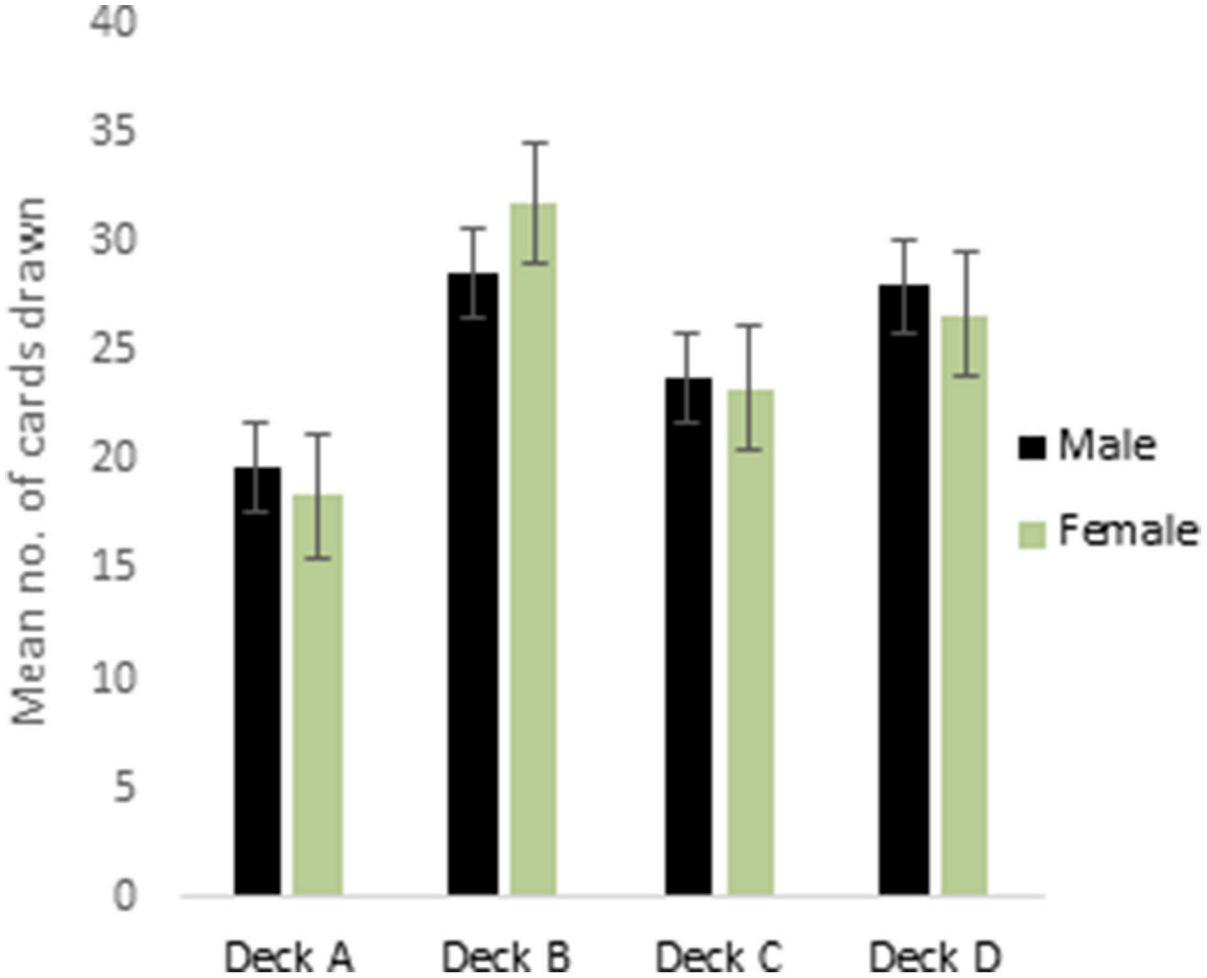
**Cards drawn by males and females from the IGT reward decks (more cards drawn from decks C and D reflect advantageous decision-making)**. Error bars represent the standard error of the mean.

**Figure 2:**
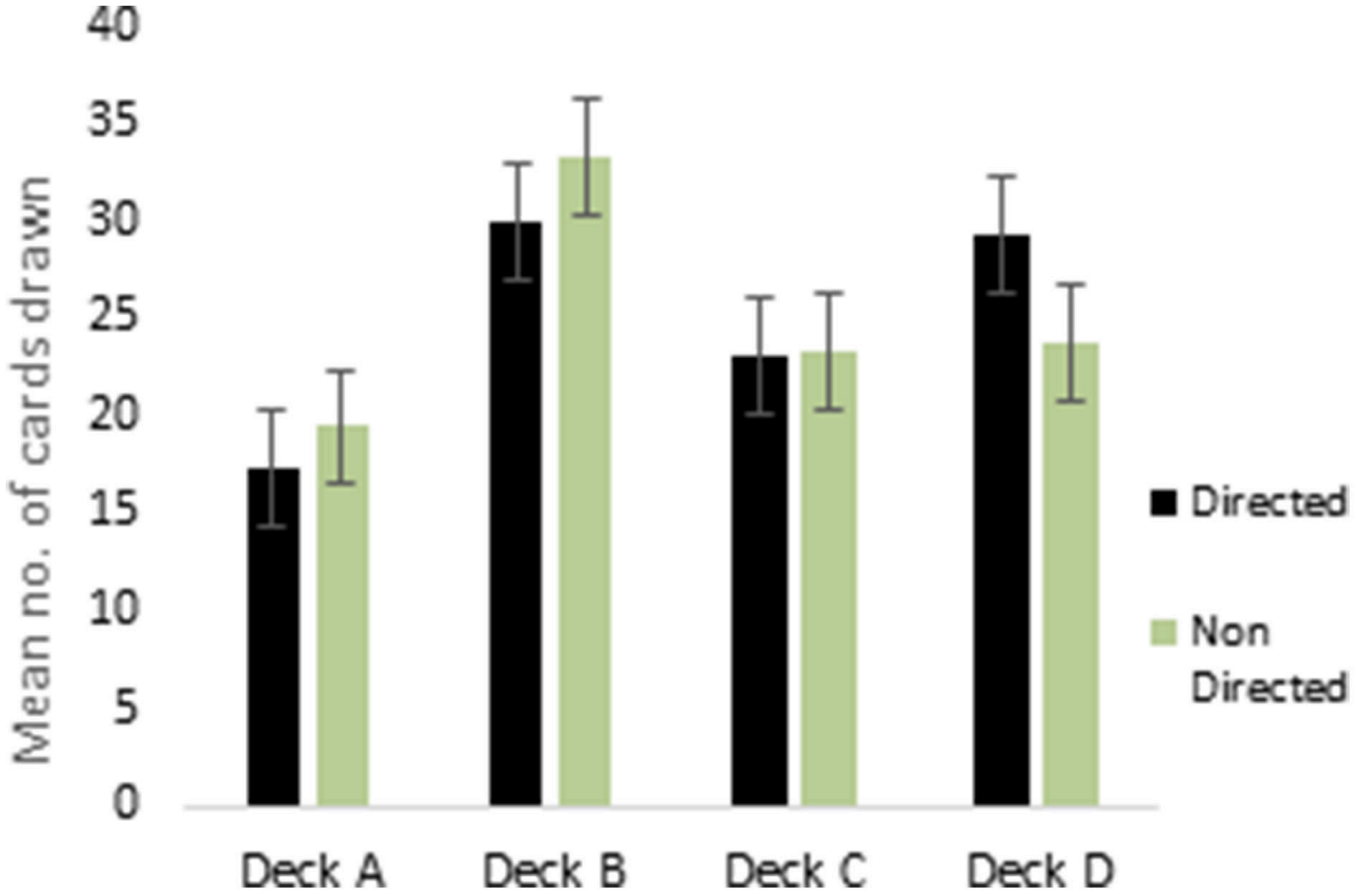
**Interaction of instruction (valence-directed instruction—“Directed”; instructions not directed toward valence—“Non Directed”) and deck choices (decks C and D are advantageous decks) in reward IGT decks for females**. Error bars represent the standard error of the mean.

ANOVA performed on the 4 decks of reward IGT showed a main effect of deck types [*F*_(2.75, 429.73)_ = 16.41, *p* < 0.01; means: deck A′ = 19.84, deck B′ = 28.63, deck C′ = 23.78, deck D′ = 27.76; Figure 1], all interactions were non-significant. Males showed differentiation between the 4 decks, but neither right-handedness nor valence-directed instructions made any contribution to deck preferences in male participants. Females showed a main effect of deck types [*F*_(2.62, 408.68)_ = 38.43, *p* < 0.01; means: deck A′ = 18.63, deck B′ = 31.71, deck C′ = 23.28, deck D′ = 26.64], and 2-way interaction of instruction and deck type was significant [*F*_(2.62, 408.38)_ = 4.78, *p* < 0.01]. Valence-directed instruction helped females choose more cards from the good deck D′ (mean = 29.45) and fewer cards from the risky deck B′ (mean = 30.06) as compared to females who had not received valence-directed instructions, who picked fewer cards from deck D′ (mean = 23.84) and more cards from deck B′ (mean = 33.36; Figure 2).

**Figure 3:**
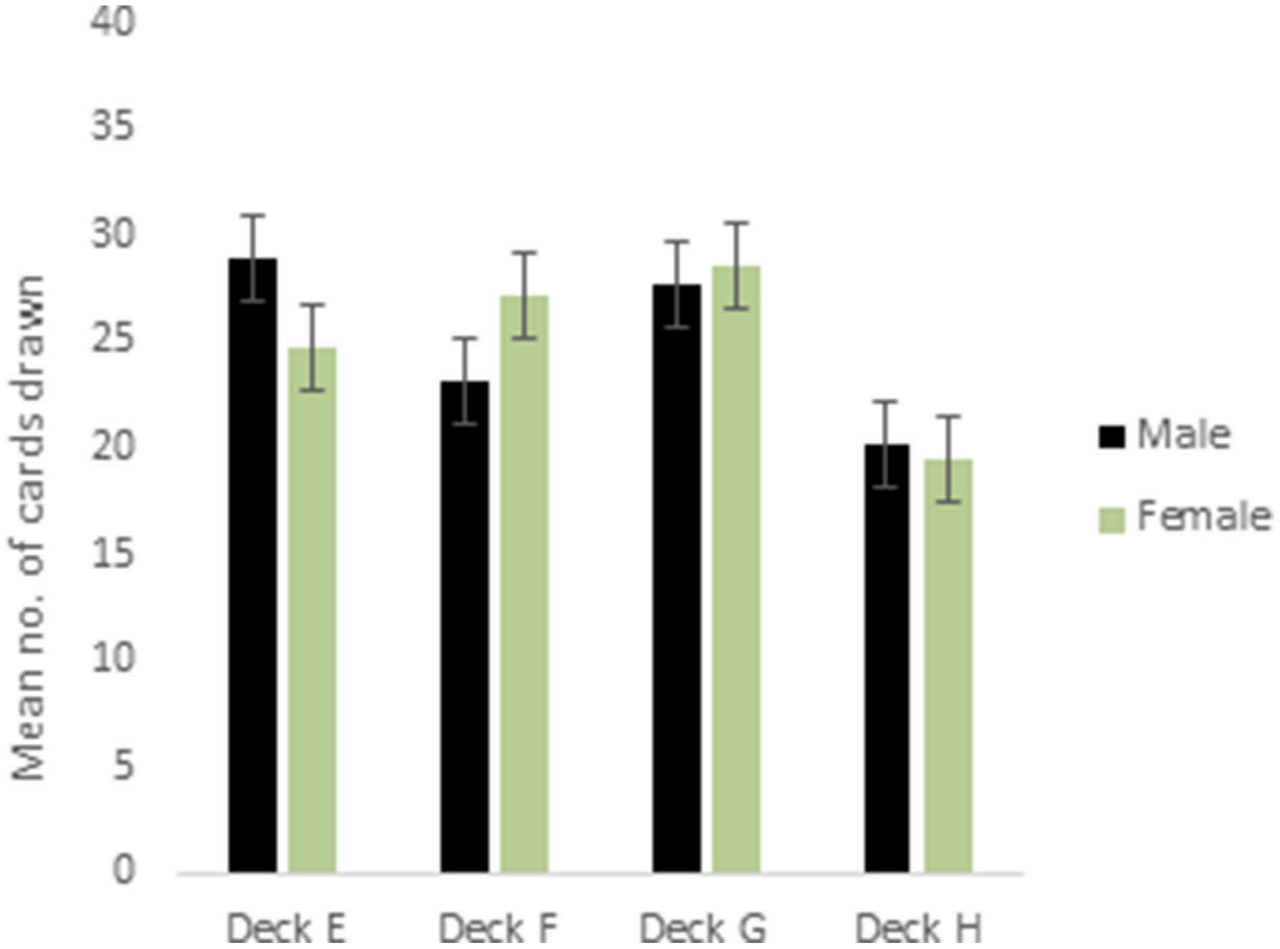
**Cards drawn by males and females from the IGT punishment decks (more cards drawn from decks E and G reflect advantageous decision-making)**. Error bars represent the standard error of the mean.

**Figure 4:**
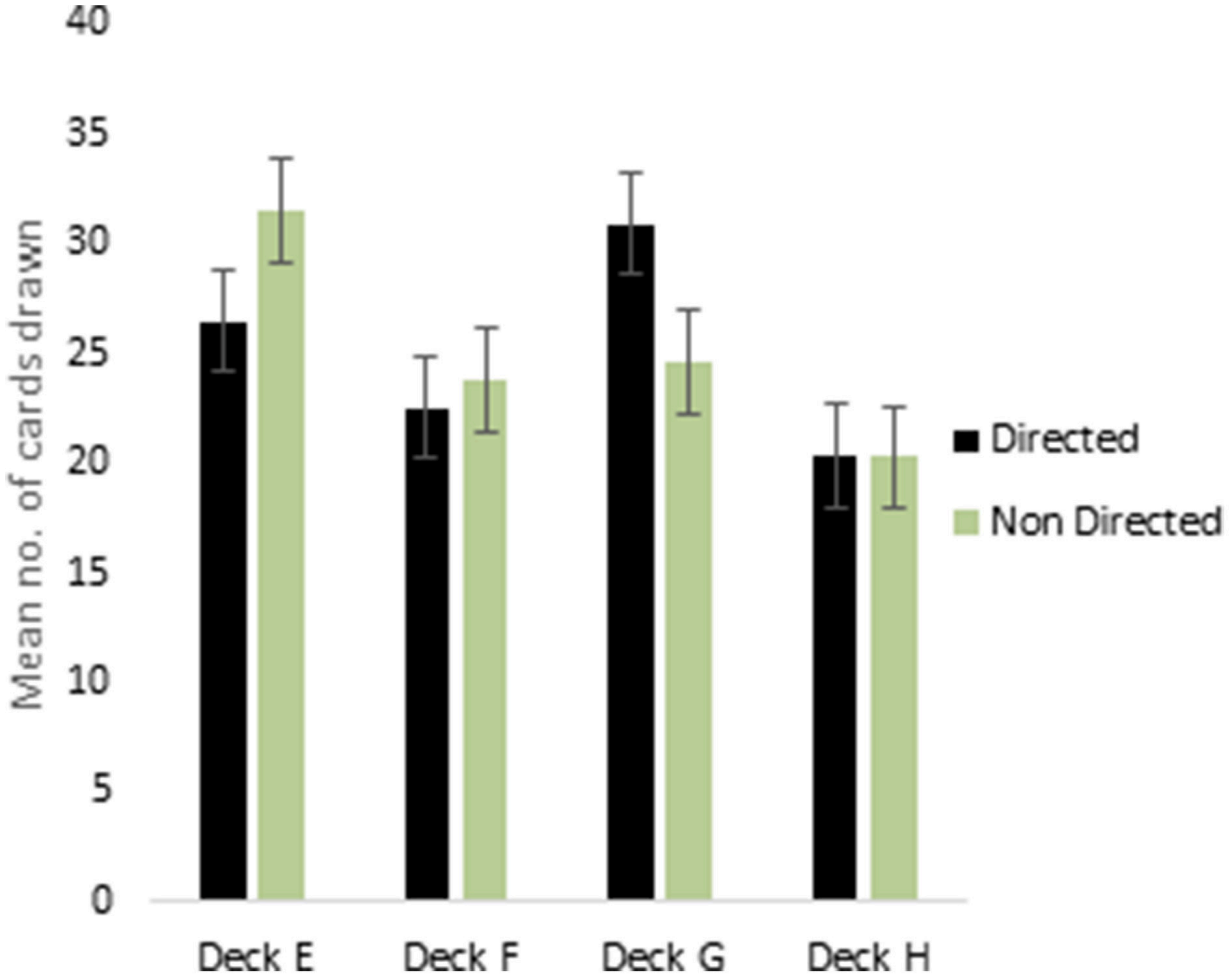
**Males showed significant interaction of instruction and deck type in punishment IGT**. Error bars represent the standard error of the mean.

ANOVA for the decks in the punishment IGT for males (i.e., decks E′, F′, G′, and H′) showed a main effect of deck types [*F*_(2.68, 418.72)_ = 10.96, *p* < 0.01; means: deck E′ = 28.41, deck F′ = 23.36. deck G′ = 27.66, deck H′ = 20.58; see Figure 3], and 2-way interaction of valence-directed instructions and deck types was significant [*F*_(2.68, 418.72)_ = 3.73, *p* < 0.05]. In males, the 4 decks were differentiated, and males who had received valence-directed instructions drew more cards from the advantageous deck G′ (mean = 30.85) than males who had received non-directed instructions (mean = 24.57; Figure 4). On the other hand, females showed a main effect of deck type [*F*_(2.69, 420.42)_ = 14.50, *p* < 0.01; means: deck E′ = 24.83, deck F′ = 27.15, deck G′ = 28.62, deck H′ = 19.40], but all interactions were non-significant. In females, the punishment IGT decks could be differentiated, but the deck choices of females remained independent of right-handedness or valence-directed task motivation (see Table 4).

**Table 4:** **Result of ANOVA for decks in the reward and the punishment IGT**.

**Sex**	**IGT type**	**Source**	***F* (df1, df2)**	***p*-value**
Male	Reward	Deck type	(2.75, 429.73) = 16.41	0.000
		Deck type × RH type	(2.75, 429.73) = 0.37	0.758
		Deck type × Instruction type	(2.75, 429.73) = 0.34	0.781
		Deck type × RH type × Instruction type	(2.75, 429.73) = 0.18	0.898
Female	Reward	Deck type	(2.62, 408.68) = 38.43	0.000
		Deck × RH type	(2.62, 408.68) = 0.18	0.888
		Deck × Instruction type	(2.62, 408.68) = 4.78	0.004
		Deck type × RH type × Instruction type	(2.62, 408.68) = 1.49	0.220
Male	Punishment	Deck type	(2.68, 418.72) = 10.96	0.000
		Deck × RH type	(2.68, 418.72) = 0.68	0.546
		Deck × Instruction type	(2.68, 418.72) = 3.73	0.015
		Deck type × RH type × Instruction type	(2.68, 418.72) = 1.03	0.372
Female	Punishment	Deck type	(2.69, 420.42) = 14.50	0.000
		Deck × RH type	(2.69, 420.42) = 1.11	0.343
		Deck × Instruction type	(2.69, 420.42) = 2.20	0.094
		Deck type × RH type × Instruction type	(2.69, 420.42) = 2.09	0.107

“*RH type” denotes right-handedness*.

**Figure 5:**
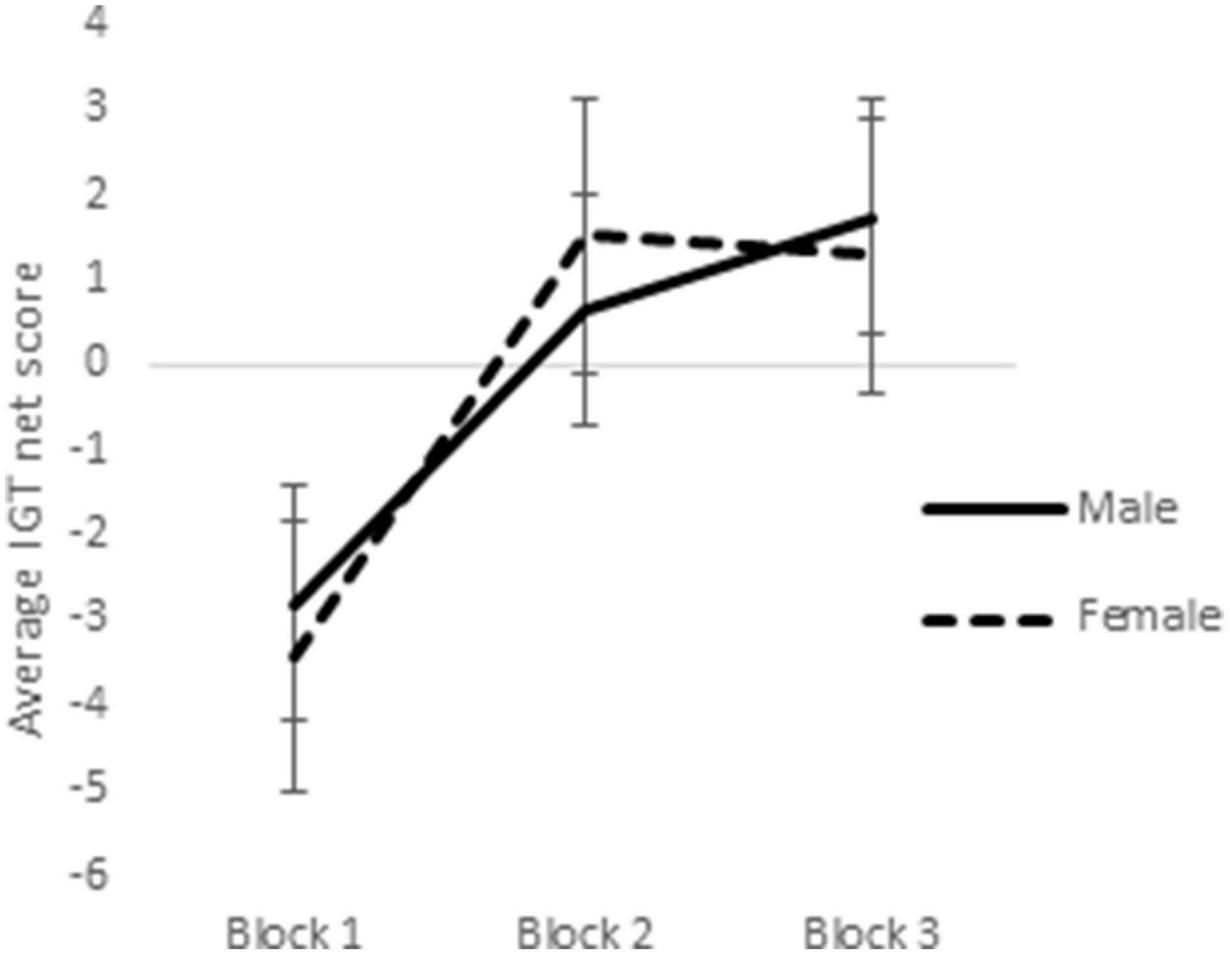
**Increases in advantageous decision-making (net scores) across blocks 1, 2, and 3 of the reward IGT shown by males and females**. Error bars represent the standard error of the mean.

**Figure 6:**
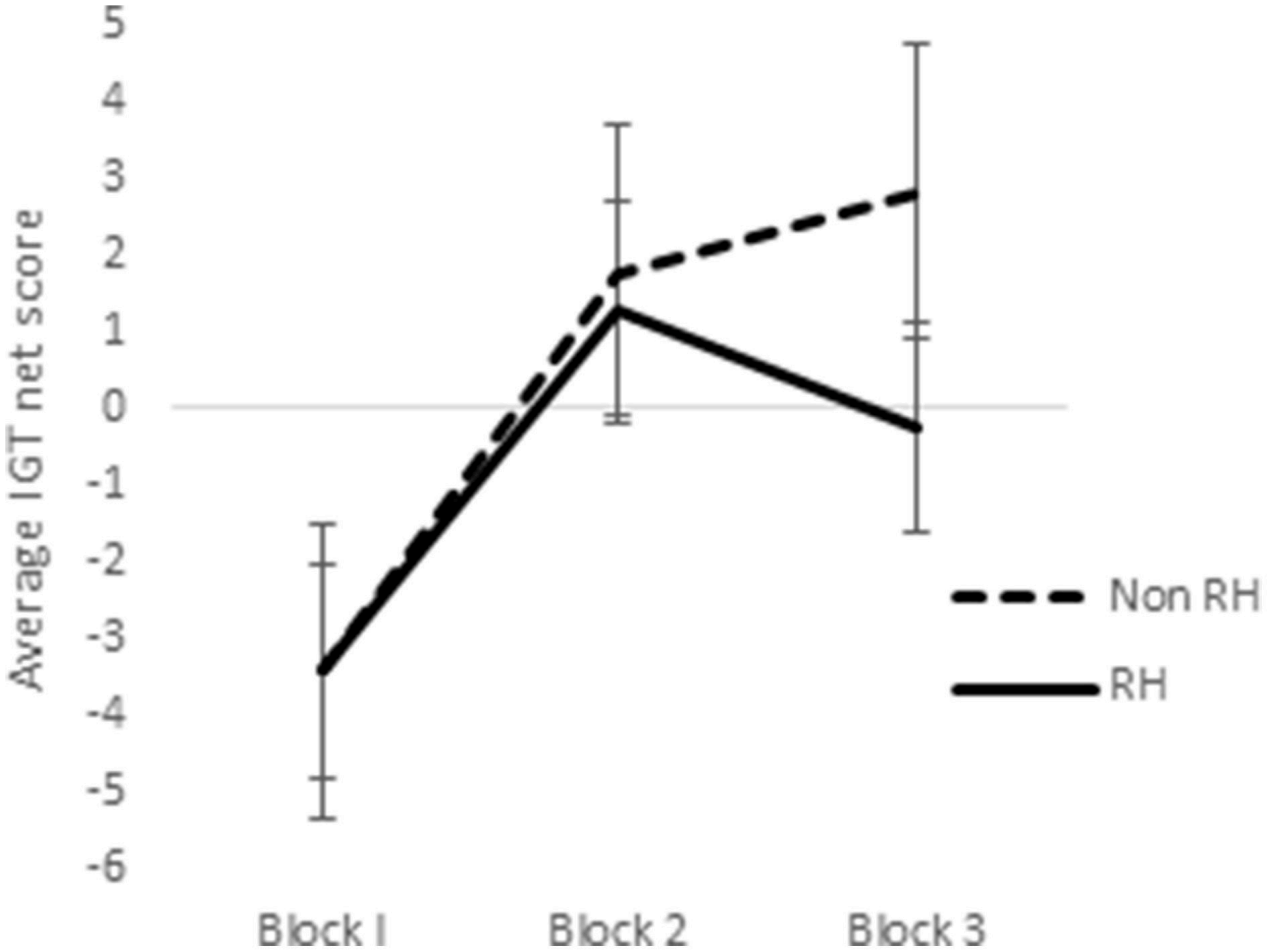
**Interaction of right-handedness (RH) and IGT reward blocks in females**. Error bars represent the standard error of the mean.

**Figure 7:**
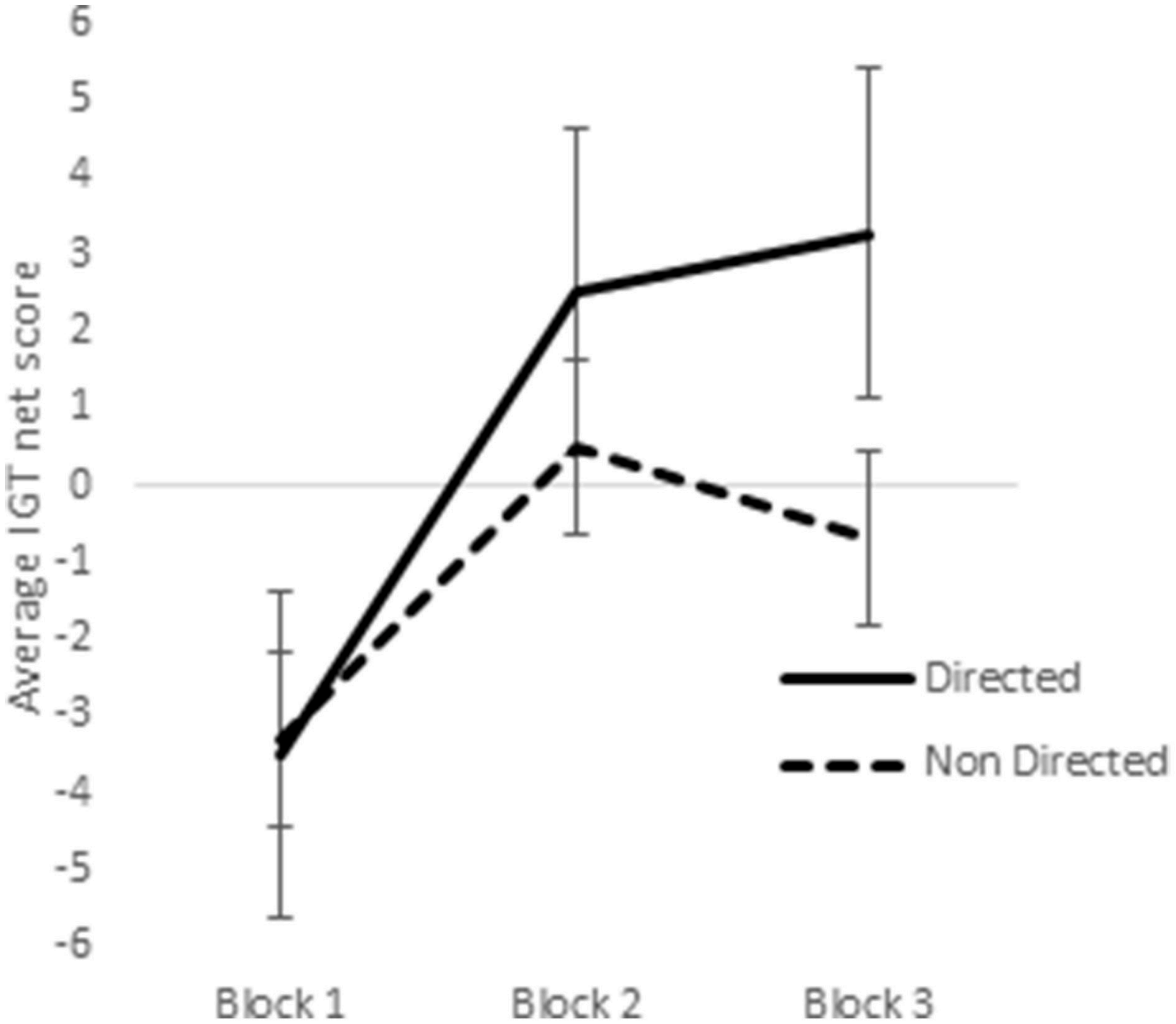
**Interaction of instruction and early blocks in the reward IGT for females**. Error bars represent the standard error of the mean.

In a previous study utilizing a sample of right-handed males and females, it was observed that sex-differences emerged in the earlier blocks of trials in the reward IGT, such that men's decision-making improved much earlier (i.e., in blocks 1 and 2) than in women (Bolla et al., 2004). Therefore, we analyzed learning across the 3 blocks of IGT. Scores on the first 3 blocks of trials (trials 1–20, 21–40, and 41–60) were analyzed by ANOVA to understand whether sex-differences in right-handedness and in valence-directed task motivation contributes to sex-differences in learning advantageous decision-making in the IGT. There was a main effect of the blocks in males [*F*_(1.84, 287.03)_ = 21.15, *p* < 0.01; means: block 1 = −2.69, block 2 = 0.65, block 3 = 1.55; Figure 5], none of these interactions were significant. Males learned across the 3 blocks of trials, independent of right-handedness and valence-directed instructions. On the other hand, females' decision-making was improved across the 3 blocks of trials [*F*_(1.82, 283.80)_ = 40.04, *p* < 0.01; means: block 1 = −3.41, block 2 = 1.51, block 3 = 1.24], and 2-way interaction of blocks and right-handedness was significant [*F*_(1.82, 283.80)_ = 3.54, *p* < 0.05; Figure 6], and interaction of blocks and valence-directedness of task instruction was significant [*F*_(1.82, 283.80)_ = 5.56, *p* < 0.01; see Figure 7]. Right-handedness and valence-directed instruction had an effect on learning in the early blocks of reward IGT for non-right-handed (means: block 1 = −3.40, block 2 = 1.77, block 3 = 2.86), rather than right-handed females (means: block 1 = −3.41, block 2 = 1.28, block 3 = −0.27), and females receiving valence-directed instructions (means: block 1 = −3.49, block 2 = 2.53, block 3 = 3.23), rather than non-directed instructions (means: block 1 = −3.33, block 2 = 0.50, block 3 = −0.75) made more advantageous decisions in the early blocks of reward IGT in females.

**Figure 8:**
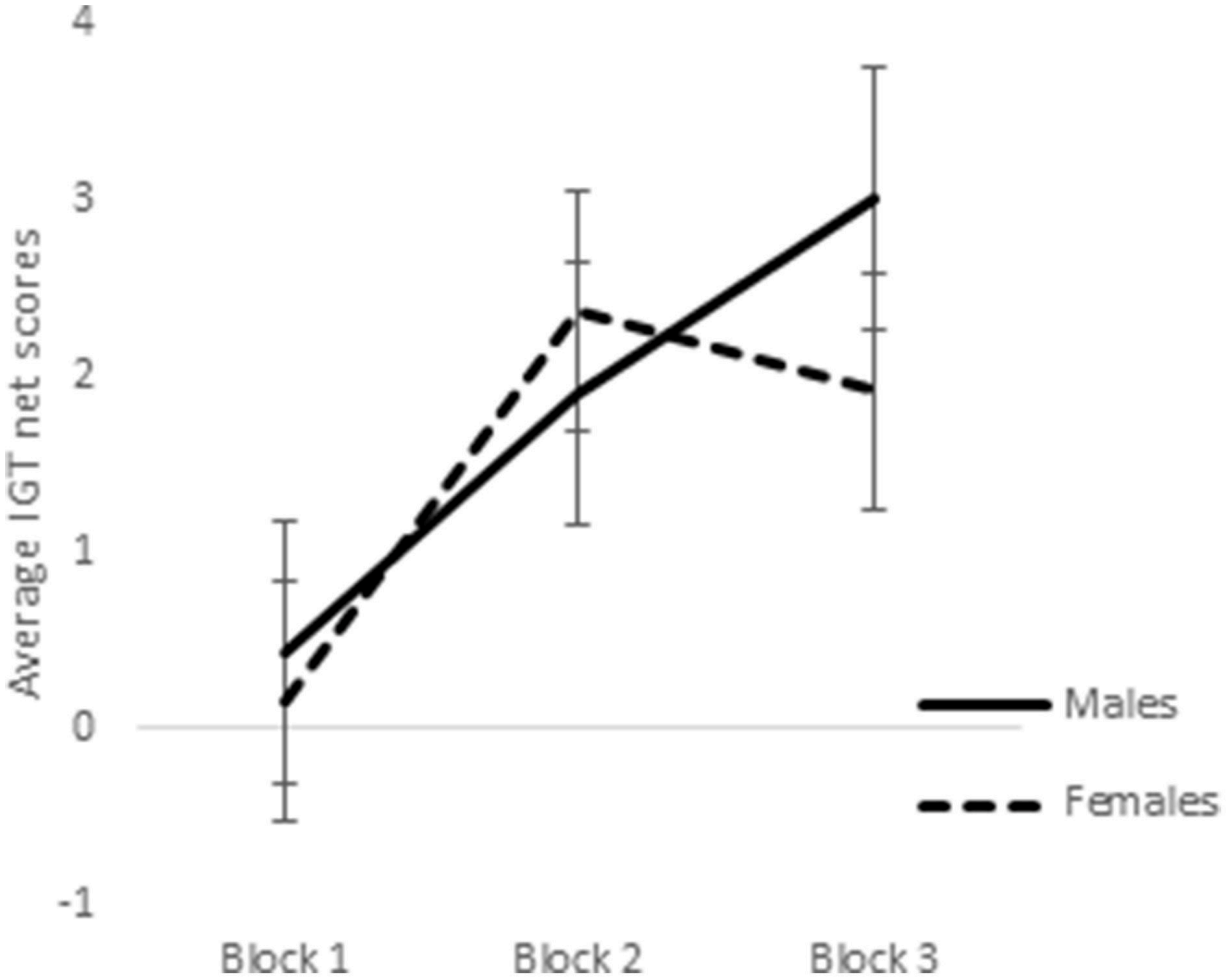
**Block-wise net scores of the punishment IGT in males and females**. Error bars represent the standard error of the mean.

**Figure 9:**
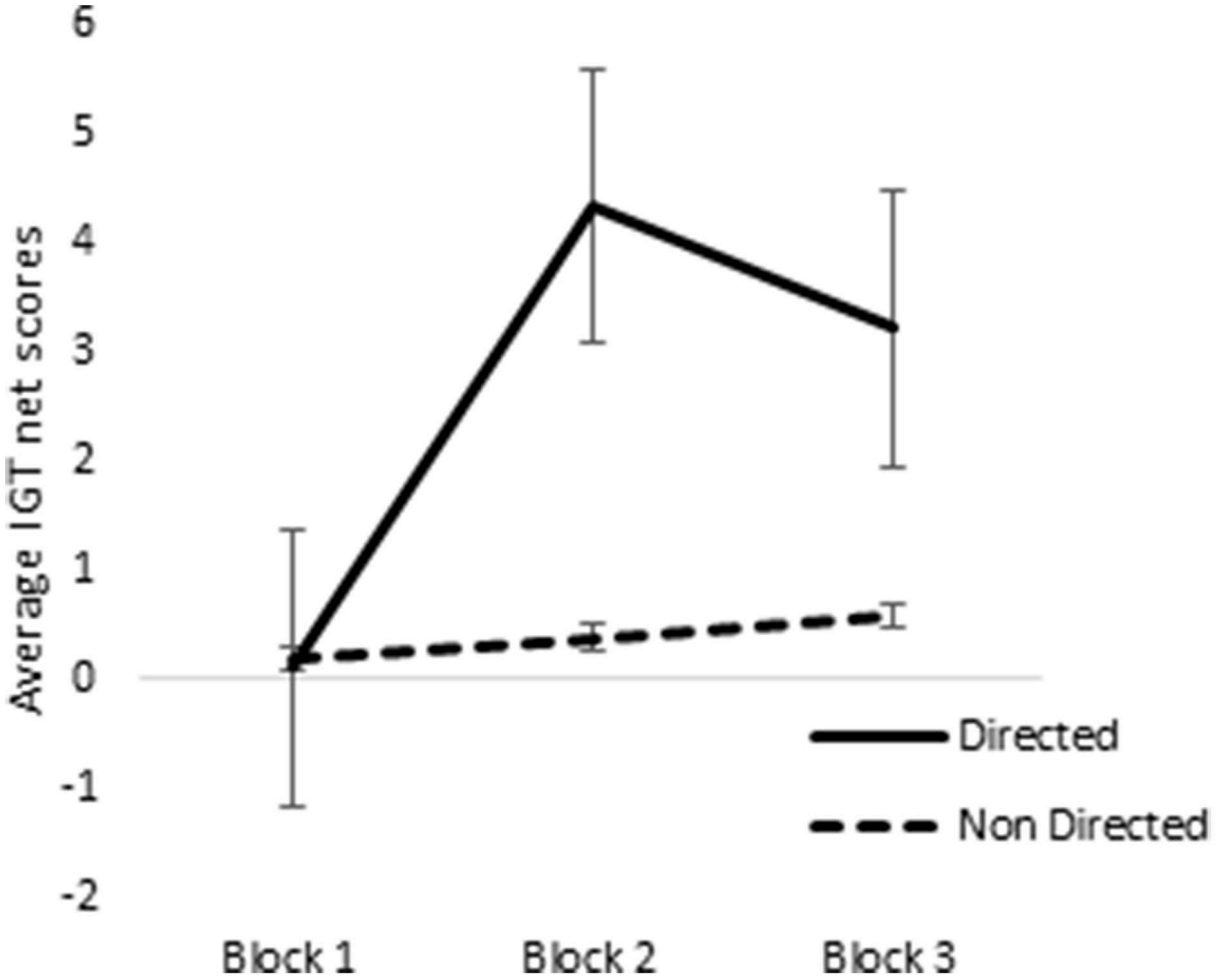
**Interaction of instruction and IGT punishment blocks in females**. Error bars represent the standard error of the mean.

ANOVA of blocks 1, 2, and 3 of punishment IGT data in male participants showed a main effect of IGT blocks [*F*_(2, 312)_ = 6.58, *p* < 0.01; means: block 1 = −2.69, block 2 = 0.65, block 3 = 1.55; see Figure 8], but none of the interactions were significant. Male participants showed an increase in advantageous decision-making in the early blocks of punishment IGT, independent of right-handedness and the valence-directedness of instructions. In females, advantageous decision-making differed across the 3 blocks of trials [*F*_(2, 312)_ = 8.88, *p* < 0.01], suggesting an increase in advantageous choices from block 1 to block 2 (means: block 1 = −3.41, block 2 = 1.51, block 3 = 1.24). Two-way interaction of the instruction and blocks was significant [*F*_(2, 312)_ = 6.85, *p* < 0.01], suggesting that females who had received valence-directed task instruction (means: block 1 = 0.13, block 2 = 4.38, block 3 = 3.25) made more advantageous decisions in punishment IGT than females who had received non-directed instructions (means: block 1 = 0.23, block 2 = 0.43, block 3 = 0.60; see Figure 9; refer to Table 5 for results of ANOVAs).

**Table 5:** **Result of ANOVAs for the early blocks of the reward and punishment IGT (blocks 1, 2, and 3 as within-subject variable)**.

**Sex**	**IGT type**	**Source**	***F* (df1, df2)**	***p*-value**
Male	Reward	Blocks (1, 2, and 3)	(1.84, 287.03) = 21.15	0.000
		Blocks × RH type	(1.84, 287.03) = 0.91	0.395
		Blocks × Instruction type	(1.84, 287.03) = 1.04	0.351
		Blocks × RH type × Instruction type	(1.84, 287.03) = 1.06	0.344
Female	Reward	Blocks (1, 2, and 3)	(1.82, 283.80) = 40.04	0.000
		Blocks × RH type	(1.82, 283.80) = 3.54	0.034
		Blocks × Instruction type	(1.82, 283.80) = 5.56	2.006
		Blocks × RH type × Instruction type	(1.82, 283.80) = 0.67	0.497
Male	Punishment	Blocks (1, 2, and 3)	(2, 312) = 6.58	0.002
		Blocks × RH type	(2, 312) = 0.18	0.837
		Blocks × Instruction type	(2, 312) = 1.05	0.350
		Blocks × RH type × Instruction type	(2, 312) = 0.01	0.990
Female	Punishment	Blocks (1, 2, and 3)	(2, 312) = 8.88	0.000
		Blocks × RH type	(2, 312) = 0.37	0.693
		Blocks × Instruction type	(2, 312) = 6.85	0.001
		Blocks × RH type × Instruction type	(2, 312) = 0.35	0.708

“*RH type” denotes right-handedness*.

**Figure 10:**
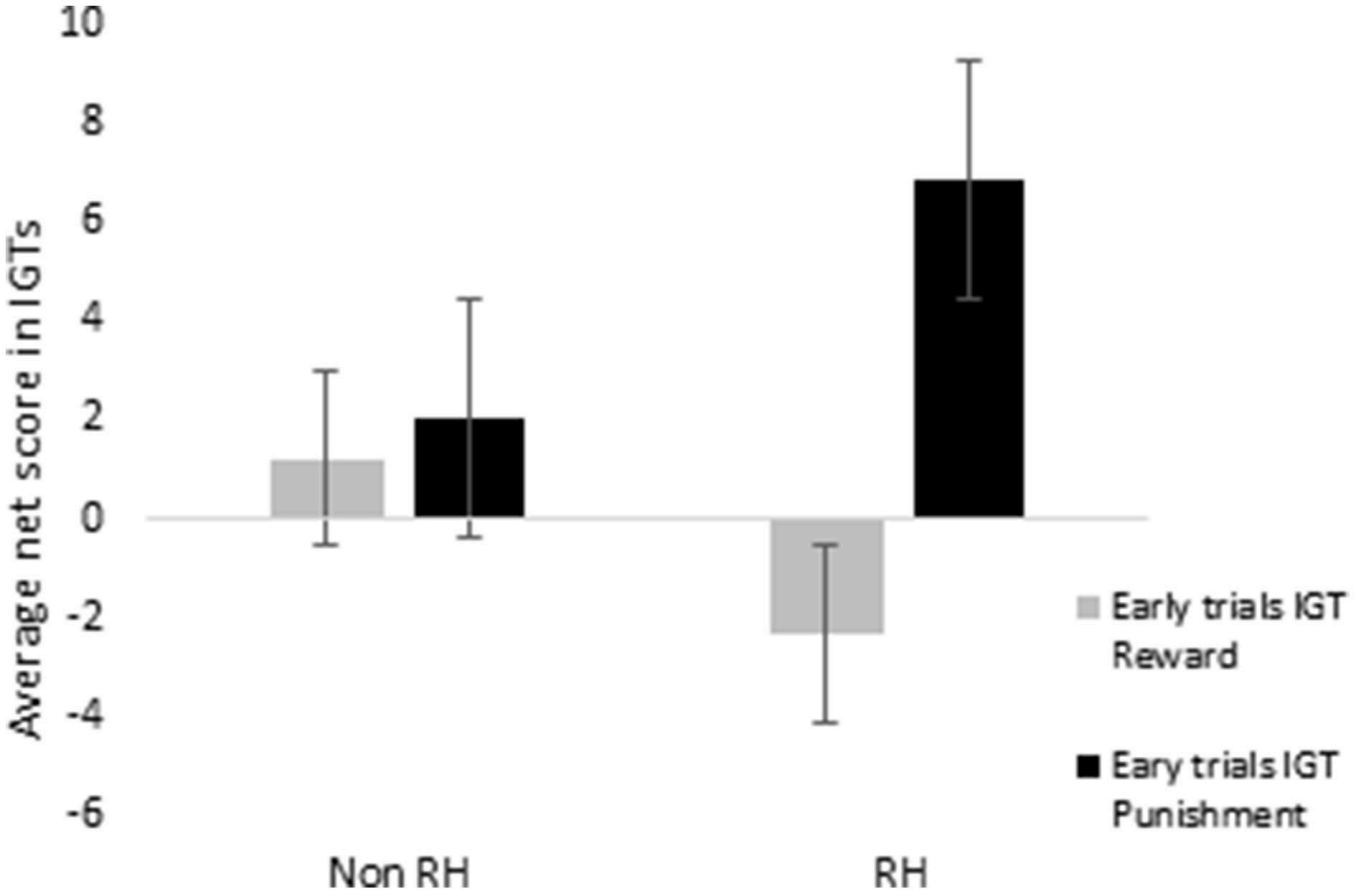
**Right-handedness (right-handed—“RH”; non-right-handed—“Non-RH”) in females showed a significant effect on early blocks of trials (i.e., total net scores of blocks 1, 2, and 3), specifically a disadvantage in the reward IGT**. Error bars represent the standard error of the mean.

When scores of blocks 1, 2, and 3 were separately totaled for reward and for punishment IGTs and evaluated by ANOVA, a main effect of IGT type was significant for males, suggesting that males showed a different rate of learning across the 2 IGTs [*F*_(1, 156)_ = 6.46, *p* < 0.05; means: reward IGT = −0.49, punishment IGT = 5.08]. None of the interactions were significant, suggesting that neither right-handedness nor task instructions contributed to differences in learning across the 2 types of IGT. On the other hand, females showed a main effect of IGT type, suggesting that learning in the early blocks differed across the 2 IGTs [*F*_(1, 156)_ = 8.55, *p* < 0.01; means: reward IGT = −0.66, punishment IGT = 4.50]. Two-way interaction of IGT type and right-handedness was significant [*F*_(1, 156)_ = 5.93, *p* < 0.05]; non-right-handed females made more advantages decisions in punishment IGT (mean = 2.05) than in reward IGT (mean = 1.22); however, right-handed females performed poorly in reward IGT (mean = −2.40), but made more advantageous decisions in punishment IGT (mean = 6.77; Figure 10).

**Figure 11:**
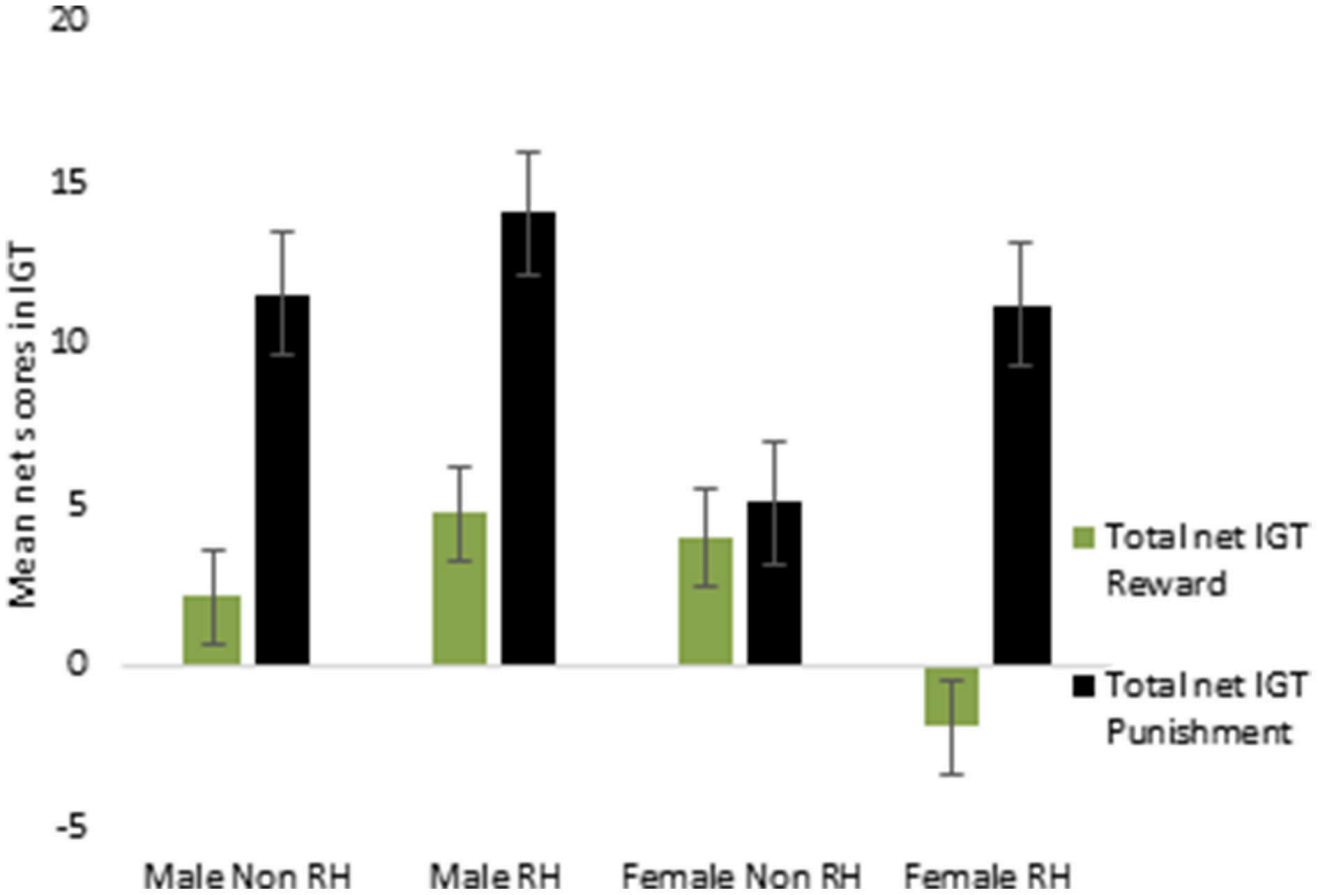
**Interaction of right-handedness (RH) and the total net IGT (total of blocks 1, 2, 3, 4, and 5) scores in females suggested that right-handed individuals made fewer advantageous decisions in the reward IGT**. Error bars represent the standard error of the mean.

Lastly, the total net scores across the 5 blocks were calculated separately for the reward and for the punishment IGT and were then investigated by ANOVA. A main effect of IGT type [*F*_(1, 156)_ = 7.44, *p* < 0.01] was significant for males (means: reward IGT = 3.06, punishment IGT = 12.24), all interactions were non-significant. This suggests that advantageous decision-making by males differed across reward and punishment IGTs, independent of right-handedness and valence-directed instructions. On the other hand, there was a main effect of IGT type for females [*F*_(1, 156)_ = 5.82, *p* < 0.05; means: reward IGT = 0.89, punishment IGT = 8.20], and the interaction of the total net scores and right-handedness was significant [*F*_(1, 156)_ = 4.14, *p* < 0.05], wherein non-right-handed females made more advantageous decisions in the punishment IGT (mean = 5.09) than in the reward IGT (mean = 4.03). In contrast, right-handed females performed poorly in the reward IGT (mean = −2.01), but performed very well in the punishment IGT (mean = 11.08; see Figure 11; refer to Table 6 for results of ANOVAs).

**Table 6:** **Result of ANOVAs for the total of early blocks, and total of 5 blocks of reward and punishment IGT**.

**Sex**	**Analysis**	**Source**	***F* (df1, df2)**	***p*-value**
Male	Total blocks 1–3	IGT type (total blocks 1, 2, 3 of reward vs. punishment)	(1, 156) = 6.46	0.012
		IGT type × RH type	(1, 156) = 0. 23	0.635
		IGT type × Instruction type	(1, 156) = 0.15	0.699
		IGT type × RH type × Instruction type	(1, 156) = 0.16	0.694
Female	Total blocks 1–3	IGT type (total blocks 1, 2, 3 of reward vs. punishment)	(1, 156) = 8.55	0.004
		IGT type × RH type	(1, 156) = 5.93	0.016
		IGT type × Instruction type	(1, 156) = 0.04	0.838
		IGT type × RH type × Instruction type	(1, 156) = 0.26	0.608
Male	Total net score	IGT type (total net score of reward vs. total net score of punishment IGT)	(1, 156) = 7.44	0.007
		IGT type × RH type	(1, 156) = 0.00	0.991
		IGT type × Instruction type	(1, 156) = 0.23	0.633
		IGT type × RH type × Instruction type	(1, 156) = 0.23	0.634
Female	Total net score	IGT type (total net score of reward vs. total net score of punishment IGT)	(1, 156) = 5.82	0.017
		IGT type × RH type	(1, 156) = 4.14	0.044
		IGT type × Instruction type	(1, 156) = 0.13	0.721
		IGT type × RH type × Instruction type	(1, 156) = 0.99	0.321

“*RH type” denotes right-handedness*.

## Discussion

The study was aimed at understanding the relationship between sex, motor, and affective lateralization in the IGT decision-making, specifically whether sex-differences in advantageous decision-making in the IGT is influenced by sex-differences in motor laterality (i.e., right-handedness) and affect laterality (i.e., valence-directed instruction and IGT type). It has been believed that advantageous IGT decision-making reflects lateralized cognitive control, and that motor and affect lateralization would benefit advantageous IGT decision-making. It was hypothesized that strong right-handedness (motor lateralization), affect or valence-directed task instructions (lateralized motivation), and a punishment frame of the IGT (affect lateralization) would facilitate advantageous IGT decision-making, such that the overall more-lateralized male sex would benefit more from lateralized constructs. Advantageous IGT decision-making was analyzed on the basis of deck choices, as well as on learning across blocks of trials. To the best of our knowledge, this is the first study to consider the role of lateralized constructs, such as right-handedness and affect, in sex-differences in the IGT decision-making in both reward and punishment IGTs. We questioned whether there is a right-handed male-advantage in IGT, and whether it differed across reward and punishment IGTs.

Correlation analysis suggested that, as the degree of right-handedness increases, there is an increase in advantageous decision-making in punishment IGT, once the effect of sex and task instruction are accounted for. In line with the contention that IGT decision-making reflects right-lateralized cognitive control, and the negative valence of punishment IGT being largely right-lateralized, laterality was expected to benefit from a punishment frame, and results in higher advantageous decision-making in the punishment IGT. Next, a series of ANOVAs were performed on data that were split by sex using either IGT decks or blocks of IGT trials to understand advantageous decision-making in reward and punishment IGTs, to test whether right-handedness and valence-directedness of task motivation, as measures of motor and affect laterality, respectively, contribute to advantageous decision-making. Both males and females differentiated between the 4 deck choices of the reward IGT; however, only females benefited from valence-directed task instructions by choosing more from the advantageous decks. The poor performance of females in the reward IGT has been attributed to female preference for the disadvantageous deck B (Overman and Pierce, 2013), and the present results suggests that females who received valence-directed instructions chose less from deck B than did females who received non-directed instructions. There are two explanations for why valence-directed females succeeded in avoiding deck B. (a) It is possible that, for females who received non-directed instructions, deck B, which carries large immediate rewards and infrequent but large losses, seemed ideal for pursuing the twin-goals of seeking rewards as well as avoiding punishments; this pursuit of twin goals might have triggered non-lateralized or bilateral activity. (b) It is possible that females who received valence-directed instructions were relieved of some of those demands by pursuing either rewards or avoiding punishments, thereby triggering lateralized activity (either right-lateralized activity in avoiding punishment or left-lateralized activity in seeking rewards). Such lateralized activity is thought to be conducive to cognitive control, resulting in better advantageous decision-making.

In the punishment decks, both males and females differentiated between the 4 decks; however, only males seemed to benefit from valence-directed instructions. These results highlight sex-differences in the IGT, and show that females relied on valence-directed task instruction for choosing good decks in the reward IGT, whereas males relied on task instructions for choosing advantageous decks in the punishment IGT. To an extent, this sex difference in reliance on valence directed-instructions might reflect sex-differences in reward and punishment sensitivity, as females tend to be reward-focused, while males tend to be sensitive to losses (Bolla et al., 2004; Evans and Hampson, 2015). Therefore, receiving valence-directed instructions may have benefited females in the reward IGT, which is focused on rewards, whereas valence–directed instructions would have benefitted males in the punishment IGT, which is focused on punishments. Unlike the reward IGT, where sex-differences in deck choices have been analyzed in detail, deck choices in the punishment IGT have rarely been discussed. Future studies aimed at attributing sex-differences in IGT-related decision-making to reward–punishment sensitivity should compare decision-making in both the IGTs.

To understand how IGT decision-making evolves with time and practice across trials, and particularly to test whether sex-difference emerge in early trials of IGT, and whether right-handedness and valence-directedness contributes thereto, analysis of the first 3 blocks of IGT trials was undertaken separately for the reward and for the punishment IGT. In the reward IGT, advantageous decision-making changed across the first 3 blocks of the reward IGT in both the sexes. These findings contradict those of a previous study in which males showed an increase in advantageous decisions in block 1 and 2, whereas females failed to show similar learning across the blocks (Bolla et al., 2004). However, these contradictory results might be due to differences in sample size and characteristics; the sample recruited by Bolla et al. (2004) was a smaller sample of right-handed, and slightly older males (*n* = 10; mean age: 32.6 years) and females (*n* = 10; mean age: 27.5 years), admitted in an in-patient facility for studying neurological differences by means of brain imaging (PET). Although both the sexes learned to make advantageous decisions in the early blocks of the reward IGT in the present study, there were sex-differences in the factors that influenced the rate of learning. Specifically, valence-directed instructions and right-handedness influenced the rate of learning in the reward IGT in females. Interestingly, when comparing non-right handed females with right-handed females, non right-handers made more advantageous decisions in the first 3 blocks of trials whereas males' performance increased across the early blocks of the reward IGT, irrespective of right-handedness or of task instructions. This sex-difference may be due to right-handedness being associated with restricted access to the right hemisphere (Propper et al., 2012), and since cognitive control underlying advantageous decision-making in the IGT is believed to be right-lateralized (Garavan et al., 1999; Aron et al., 2003, 2004; Knoch et al., 2006), it is possible that this restricted right-hemispheric access due to right-handedness is more detrimental to females than to males. Furthermore, valence-directed instructions influenced the rate of learning in the early blocks of the reward IGT in females. As mentioned earlier, valence-directed instructions might trigger affect-related motivation, which is lateralized, more so than non-directed instructions, which might lack affect-directedness and hence trigger bilateral or non-lateralized activity, thereby being detrimental for cognitive control and advantageous decision-making. It is also possible that there were no sex differences in the early blocks of the reward IGT, because half of the female sample received valence-directed instructions. Future studies should utilize affect-directed instructions to determine whether the results can be replicated, and whether improvement occurs in female advantageous decision-making in the reward IGT.

Advantageous decision-making changed across the 3 blocks of punishment IGT in both the sexes. Even though punishment IGT is rarely used, the rate of learning seemed to have shown improvement across the early trials in other studies (e.g., Bechara et al., 2000; Must et al., 2006, 2007; Verdejo-Garcia et al., 2007). Females who received valence-directed instructions showed greater learning than females who received non-directed instructions. It is interesting that, even though males benefited from valence-directed instructions while choosing advantageous decks in punishment IGT, valence-directed instructions did not facilitate advantageous decision-making in the early trials of the punishment IGT for males. On the other hand, valence-directed instructions facilitated block-wise advantageous decision-making in the early blocks of both the reward and the punishment IGTs in females.

On comparing learning in the early blocks of the reward and the punishment IGTs, results suggested that both males and females showed different rates of learning across the 2 IGTs; however, interesting sex-differences emerged, as right-handedness contributed to differences in the learning observed in the 2 IGTs. More specifically, right-handed females performed poorly on the reward IGT and performed well in the punishment IGT, thereby showing prominent inconsistencies in advantageous decision-making across the reward and the punishment IGTs. On comparing the total advantageous decisions made across the 5 blocks of the reward IGT with the total advantageous decisions made in the punishment IGT, we found that right-handed females performed disadvantageously in the reward IGT, but performed advantageously in the punishment IGT. Advantageous decisions in the early blocks made by males differed across the reward and punishment IGTs; however, this difference was independent of right-handedness or the instructions given. Assuming that reward and punishment is lateralized, it was expected that valence-directed task instructions that are solely directed toward reward or punishment would benefit advantageous decision-making by triggering much more lateralized activity than non-directed instructions. Accordingly, valence-directed instructions, as a measure of affect laterality, facilitated advantageous decision-making in females, irrespective of whether the reward or punishment forms of the IGT were used, and therefore did not contribute to frame-induced inconsistency in advantageous decision-making across the 2 IGTs. In contrast, right-handedness in females resulted in a selective disadvantage in the widely used reward IGT, and thereby contributed to inconsistent advantageous decision-making across the reward and punishment IGTs. These results add to our understanding of the role of valence-directed motivation in IGT decision-making; it was previously observed that the instruction to seeks reward benefitted advantageous decision-making selectively in the punishment IGT (Singh and Khan, 2012), facilitated separating long-term decision-making from frequency-based decision-making selectively in the reward IGT (Singh, 2013), and in the present study it was observed that both types of valence-directed instructions (i.e., only seeking reward, or only avoid punishment) facilitated advantageous decision-making, irrespective of the IGT frame, as compared to the 2 non-valence directed instructions (seeking reward, as well as avoiding punishment, or no-specific direction). Moreover, females seemed to benefit from valence-directed instructions in the early trials of both types of IGTs, probably due to markedly more lateralized activity under valence-directed motivation.

The results of this study highlight interesting similarities and dissimilarities between the sexes. The number of advantageous decisions made by both males and females differed across the reward and punishment IGTs, suggesting that both the sexes showed frame-induced inconsistencies in advantageous decision-making in the IGT, which is not triggered by the type of task motivation in either sex. However, interesting sex-differences emerged, as right-handed females performed poorly in the widely used reward IGT and performed well in the punishment IGT, whereas right-handedness did not confer such a disadvantage in males. This suggests that, since most of the IGT studies compared right-handed males with right-handed females, and excluded mixed handed or left-handed participants (e.g., Bolla et al., 2004; Fukui et al., 2005; Knoch et al., 2006; Verdejo-Garcia et al., 2007; Lawrence et al., 2009), it is possible that the right-handed female sample performed poorly in the IGT reward variant compared to the right-handed male sample. No other study had shown a right-handed disadvantage for females in the IGT context; however, a right-handed disadvantage for female participants has been observed in another task of inhibitory control, viz., the Stroop task (Beratis et al., 2010). It other words, right-handedness might contribute to the widely observed sex-differences in IGT decision-making. Since right-handedness influences sex-differences, especially in right-lateralized tasks (Crucian and Berenbaum, 1998), future studies should explore whether the IGT is a right-lateralized task, and specifically whether IGT has a “right-handed male advantage.”

Why would right-handedness matter for sex-differences in the IGT decision-making? It appears that sex- and hemispheric-differences influence decision-making across species. A male-advantage in IGT decision-making is not restricted to human IGT performance, but is also observed in rodent IGT performance (e.g., van den Bos et al., 2012). The observed sex-differences in rodent IGT has been at least partly ascribed to sex-differences in processing, namely, males show global processing, whereas females are more detail-oriented and show local processing (van den Bos et al., 2012). Since global processing is more right-lateralized (Fink et al., 1997), it implies that decision-making in males might be right lateralized, which is in line with the observation that right-lateralized behavior (i.e., behavior that is preferentially governed by the right hemisphere) is likely to show prominent sex-differences (Sullivan et al., 2014). In humans, a slight increase in the stress hormone cortisol in females seems to enhance right hemispheric activation and to result in higher advantageous decisions in the reward IGT (van den Bos et al., 2009). Interestingly, a temporary decrease in dopamine in healthy males impairs advantageous decision-making in the reward IGT (Sevy et al., 2006), and dopamine asymmetries in humans seem to alter with right-handedness, such that the right hemisphere produces relatively more dopamine (Mohr et al., 2003). The present results suggest that hemispheric differences, represented by handedness, may contribute to sex-differences in the IGT decision-making.

Sex-specific lateralization in IGT decision-making is receiving increasing attention in research (e.g., Sutterer et al., 2015). In line with the observation that hemispheric lateralization in the IGT is modulated by sex (Tranel et al., 2005), the results of the present study suggested that right-handedness contributes to sex-differences in IGT-related decision-making. Thus, sex-specific lateralization of cognitive control in the IGT may further be influenced by motor and affective lateralization. These results have to be interpreted in the light of limitations of the study, such as not accounting for disposition (Franken and Muris, 2005) and mood (Suhr and Tsanadis, 2007), which are measures associated with affect lateralization (Davidson, 1995), and are known to influence IGT decision-making. Another limitation of the study is that motor lateralization in terms of degree of handedness was not balanced in terms of sex, as noted in the literature: the left-handed population is largely male (Oldfield, 1971). Additionally, whether assessing sex-differences in the IGT, or in right-handedness, the conclusions drawn are limited by the characteristics of the tool; for example, it has been observed that certain items on the handedness inventory measure the ability to imagine carrying out an action, and hence taps into the ability to form mental images, apart from hand preference (White and Ashton, 1976). It would be interesting to test whether there are sex-differences in the ability to imagine (specifically, motor imagery), and if these differences influences sex-differences in long-term decision-making. Furthermore, response patterns on the Edinburg Inventory has produced interesting sex-differences *per se*: it has been observed that, unlike females, males hesitate to use the extreme response (males use “usually,” rather than “always”) in the rating scale of handedness and hence are more likely to be labeled as mixed-handers, even though their usage of the non-dominant hand may not be that typical (Bryden, 1977). Nevertheless, the inventory is widely used to ascertain right-handedness in most of the IGT studies. Future studies should specifically aim at ascertaining right-lateralization of IGT decision-making in healthy adults, and should ensure inclusion of a gender-balanced left- and mixed-handed sample.

Apart from showing that right-handedness accounted for sex-differences in cognitive control, the present study has added to the growing literature on the inter-relationship between different lateralized constructs; for instance, recently, it has recently been observed that the link between affect, motor, and language lateralization is clear only for right-handers (Costanzo et al., 2015). Therefore, the study is also a response to a recent call to include non-right-handed subjects in investigations targeted at understanding decision-making and lateralized constructs, such as risk, reward, and punishment (Willems et al., 2014). The results of this study also add to the body of knowledge on task-specific characteristics and their implications for our understanding of sex-differences in cognitive processing (Miller and Halpern, 2014).

## Author contributions

The author confirms being the sole contributor of this work and approved it for publication.

### Conflict of interest statement

The author declares that the research was conducted in the absence of any commercial or financial relationships that could be construed as a potential conflict of interest.

## Appendix

Two types of instructions were used in the study: (1) Valence-non-directed, and (2) Valence-directed instructions, for reward and punishment variants of the IGT.

**1a. Valence-non-directed (bidirectional instructions, reward variant):** “In front of you on the screen, there are 4 decks of cards: A′, B′, C′, and D′. When we begin the game, I want you to select 1 card at a time, by clicking on a card from any deck. Each time you select a card, the computer will tell you that you won some money. I do not know how much money you will win. You will find this out as you go along. Every time you win, the green bar at the top of the screen will become bigger. Every so often, when you click on a card, the computer will tell you that you won some money, as usual, but it will also say that you lost some money. I do not know when or how much you will lose. You will this find out as you go along. Every time you lose, the green bar at the top of the screen will become smaller. You are absolutely free to switch from 1 deck to another at any time, and as often as you wish. ***The goal of the game is to win as much money as possible and to avoid losing as much money as possible***. You will not know when the game will end. Simply keep on playing until the computer stops. You will have $2000 of credit, shown by the green bar, at the start of the game. The only hint I can give you, which is the most important thing to note, is this: out of these 4 decks of cards, some are worse than others. To win, you should try to stay away from bad decks. No matter how much you find yourself losing, you can still win the game if you avoid the bad decks. Moreover, the computer does not change the position of the decks once the game begins. It does not make you lose at random, or make you lose money based on the last card you picked.”
**1b. Reinforcement-non-directed (bidirectional instructions, punishment variant):** “In front of you on the screen, there are 4 decks of cards: E′, F′, G′, and H′. When we begin the game, I want you to select 1 card at a time by clicking on a card from any deck. Each time you select a card, the computer will tell you that you lost some money. I do not know how much money you will lose. You will find this out as you go along. Every time you lose, the green bar at the top of the screen will become smaller. Every so often, when you click on a card, the computer will tell you that you lost some money, as usual, but it will say that you gained some money as well. I do not know when or how much you will gain. You will find this out as you go along. Every time you gain some money, the green bar at the top of the screen will become bigger. You are absolutely free to switch from 1 deck to the other at any time, and as often as you wish. ***The goal of the game is to avoid losing as much money as possible and to win as much money as possible***. You will not know when the game will end. Simply keep on playing until the computer stops. You will have $2,000 of credit, shown by the green bar, at the start of the game. The only hint I can give you, which is the most important thing to note, is this: out of these 4 decks of cards, some are better than others. To win, you should try to choose from the good decks. No matter how much you find yourself losing, you can still win the game if you choose from the good decks. Moreover, the computer does not change the position of the decks once the game begins. It does not make you win or lose at random, or make you win or lose money based on the last card you picked.”
**1c. Valence-non-directed (non-directional, no-hint instructions, reward variant):** “In front of you on the screen, there are 4 decks of cards: A′, B′, C′, and D′. When we begin the game, I want you to select 1 card at a time by clicking on a card from any deck. You are absolutely free to switch from 1 deck to another at any time, and as often as you wish. You will not know when the game will end. Simply keep on playing until the computer stops. You will have $2000 of credit, shown by the green bar, at the start of the game. Moreover, the computer does not change the position of the decks once the game begins. It does not make you lose at random, or make you lose money based on the last card you picked.”
**1d. Valence-non-directed (non-directional, no-hint instructions, punishment variant):** “In front of you on the screen, there are 4 decks of cards: E′, F′, G′, and H′. When we begin the game, I want you to select 1 card at a time by clicking on a card from any deck. You are absolutely free to switch from 1 deck to another at any time, and as often as you wish. You will not know when the game will end. Simply keep on playing until the computer stops. You will have $2000 of credit, shown by the green bar, at the start of the game. Moreover, the computer does not change the position of the decks once the game begins. It does not make you lose at random, or make you lose money based on the last card you picked.”
**2a. Valence-directed (unidirectional instructions, seek reward, reward variant):** The same as in the bidirectional instructions, reward variant, except that the text in bold is changed to “*The goal of the game is to win as much money as possible*.”
**2b. Valence-directed (unidirectional instructions, seek reward, punishment variant):** The same as in the bidirectional instructions, punishment variant, except that the text in bold is changed to “*The goal of the game is to win as much money as possible*.”
**2c. Valence-directed (unidirectional instructions, avoid punishment, reward variant):** The same as in the bidirectional instructions, punishment variant, except that the text in bold is changed to “*The goal of the game is to avoid losing as much money as possible*.”
**2d. Valence-directed (unidirectional instructions, avoid punishment variant):** The same as in the bidirectional instructions, punishment variant, except that the text in bold is changed to “*The goal of the game is to avoid losing as much money as possible*.”
